# Strain-specific outer membrane vesicle-mediated transfer of a *bla*_OXA-24_-like plasmid in *Acinetobacter baumannii* restricts cross-species dissemination

**DOI:** 10.1128/msphere.00311-26

**Published:** 2026-08-20

**Authors:** Shengnan Jiang, Biyong Yan, Haiyang Liu, Long Sun, Yan Chen, Lilin Liu, Yunsong Yu

**Affiliations:** 1 Center of Laboratory Medicine, Department of Clinical Laboratory, Zhejiang Provincial People’s Hospital, People’s Hospital of Hangzhou Medical College, Hangzhou, China; 2 Zhejiang University School of Medicine 26441, Hangzhou, Zhejiang, China; 3 Department of Laboratory, The Second Affiliated Hospital Zhejiang University School of Medicine 89681, Hangzhou, China; 4 Department of Clinical Laboratory, Hangzhou Women’s Hospital (Hangzhou Maternity and Child Health Care Hospital), Hangzhou, Zhejiang, China; 5 Department of Infectious Diseases, Sir Run Run Shaw Hospital, Zhejiang University School of Medicine, Hangzhou, Zhejiang, China; 6 Department of Infectious Diseases, Sir Run Run Shaw Hospital, Alaer Hospital, Zhejiang University School of Medicine, Hangzhou, Zhejiang, China; Medical College of Wisconsin, Milwaukee, Wisconsin, USA

**Keywords:** *Acinetobacter *spp., *bla*_OXA-24_-like, outer membrane vesicles, strain specificity, cross-species barriers

## Abstract

The rapid rise of multidrug-resistant *Acinetobacter* spp. poses a major global health threat. In *Acinetobacter* spp., carbapenem resistance is frequently mediated by OXA-type carbapenemases encoded by *bla*_OXA_ genes, including *bla*_OXA-24_-like genes such as *bla*_OXA-72_. Outer membrane vesicles (OMVs) are nanoscale proteoliposomes secreted by Gram-negative bacteria that can facilitate plasmid transfer, yet the mechanisms limiting their dissemination remain unclear. Nationwide screening in China of 2,880 *Acinetobacter* clinical isolates identified 10 strains carrying *bla*_OXA-24_ and *bla*_OXA-72_ plasmids across three species (*A. baumannii*, *A. pittii*, and *A. baylyi*). Sequencing revealed nine distinct plasmids, most bearing a Rep_3/OrfX C-module backbone with *blaOXA*-p*dif* resistance modules (9/10). Among clinical strains, A2485 was identified as a high-OMV producer (2.74 ± 0.20-fold relative to ATCC 17978, *P* < 0.001). OMVs from A2485 transferred the *bla*_OXA-72_ plasmid to ATCC 17978 at 1.9 × 10⁻⁷ transformants per CFU, exceeding direct supernatant transfer at 3.2 × 10⁻⁸ transformants per CFU, and conferred resistance to meropenem, ceftazidime, and cefoperazone/sulbactam. Transfer was strictly donor-specific, with no transmission observed across 13 non-*A*. *baumannii* strains, and recipient strains failed to retransmit the plasmid, indicating a strong cross-species barrier and a non-reciprocal, donor-dependent process. Although all strains produced intact OMVs and encapsulated plasmid DNA, packaging efficiency varied considerably by host (~4.5–28.6 ng/μg). Strains with DNA loading (17978E/17978V: 28.62 and 23.98 ng/μg) equal to or exceeding that of donor A2485 (13.62 ng/μg) remained incapable of transfer. These findings demonstrate that dissemination is constrained by host-specific, post-packaging barriers rather than packaging efficiency, limiting resistance spread to specific lineages while enabling localized adaptation.

**IMPORTANCE:**

*Acinetobacter baumannii* is a leading hospital-associated pathogen, and carbapenem resistance mediated by *bla*_OXA-24_-like genes poses a serious clinical challenge. Through nationwide screening of 2,880 clinical *Acinetobacter* isolates in China, we found that strains carrying *bla*_OXA-24_ and *bla*_OXA-72_ plasmids are relatively rare, occurring in only 10 isolates across three species. We provide the first evidence that outer membrane vesicle (OMV)-mediated plasmid transfer is subject to strict, host-specific barriers: the *bla*_OXA-72_ plasmid was successfully transferred among *A. baumannii* strains via OMVs, yet no transfer was observed to any of the 13 non-*A*. *baumannii* species tested. Transfer was also non-reciprocal—recipient strains that acquired the plasmid were unable to further disseminate it, whether via OMVs or supernatants. Critically, this restriction was not explained by DNA packaging efficiency, as strains with equal or greater plasmid loading than the donor remained incapable of transmission, pointing instead to post-packaging, donor-specific factors, such as membrane composition or vesicle biogenesis machinery. These findings clarify the sporadic distribution of *bla*_OXA-24_-like plasmids in China and challenge the conventional assumption that mobile genetic elements inherently carry broad dissemination potential, revealing that OMV-mediated resistance spread is constrained to specific bacterial lineages.

## INTRODUCTION

The global increase in antimicrobial resistance poses a critical public health threat (1, 2). *Acinetobacter baumannii*, along with *A. nosocomialis* and *A. pittii*, have emerged as a leading cause of nosocomial infections (3). Carbapenem resistance in these pathogens is predominantly mediated by OXA-type carbapenemases (class D β-lactamases), among which *bla*_OXA-24_ and its variants *bla*_OXA-72_ and *bla*_OXA-139_, distinguished by specific amino acid substitutions within an otherwise conserved enzyme scaffold, are of particular concern (4–8). The OXA-24/40-like carbapenemase family was first identified in *A. baumannii* isolates from Spain, whereas OXA-72, an important variant of this family, was first reported in Thailand (9, 10). Although OXA-24/40-like enzymes were initially reported mainly in Europe, they have since been detected in Asia, South America, and other regions (9–14). In contrast, the prevalence of OXA-24 and OXA*-72* carbapenemases in *Acinetobacter* spp. is relatively low in China (15–17). Current research mainly focuses on the screening of resistance genes, with limited studies on the mechanisms of gene dissemination.

In *Acinetobacter*, the horizontal mobilization of carbapenemase genes, including *bla_OXA-24/72_*, is facilitated in part by p*dif* (plasmid *dif*) sites, which play a central role in the intra- and inter-plasmid shuffling of resistance determinants (18, 19). These 28-bp sequences are derived from the chromosomal *dif* system used for chromosome dimer resolution (20). Each *dif* module consists of a discrete DNA segment flanked by inversely oriented XerC- and XerD-binding sites separated by a 6-bp central spacer. These modules can carry diverse genetic cargos, including but not limited to antibiotic resistance determinants, and may mediate their dissemination through XerC/XerD-mediated site-specific recombination (19). The host-encoded XerC and XerD recombinases act on these sites cooperatively, mediating intramolecular inversions and intermolecular plasmid fusions and resolutions. This mechanism enables the shuffling of diverse resistance determinants, including *bla_OXA-24/72_*, *bla_OXA-58_*, *tet39*, and *msrE-mphE*, across plasmid scaffolds (18, 21, 22). Within plasmids, these modules are organized into “*dif* modules,” comprising alternating D-type and C-type modules defined by the orientation of their terminal p*dif* sites. This structural arrangement is central to the genetic plasticity and multidrug resistance evolution of *A. baumannii* (18). The plasmids harboring such module arrays have been classified within the Rep_3/OrfX replicon group, wherein the plasmid backbone functions as a structural scaffold for gene acquisition via XerC/XerD-mediated recombination (23).

Conventional horizontal gene transfer (HGT) mechanisms, such as conjugation, transduction, and transformation, do not fully account for the dissemination of non-conjugative *bla*_OXA-24_-like plasmids (24). Outer membrane vesicles (OMVs), nanoscale vesicles secreted by Gram-negative bacteria and enriched in lipids, proteins, and nucleic acids, have recently been proposed as an alternative pathway (25–27). OMVs play roles in bacterial pathogenesis, signaling, and HGT, and have been shown to mediate the transfer of *bla*_OXA-24_ and *bla*_NDM-1_ among *A. baumannii* strains (28–30). However, key questions remain unresolved: can OMVs disseminate *bla*_OXA-24_-like plasmids across species, or is transfer restricted to *A. baumannii*? Do recipient strains further propagate plasmids, or is the process strictly donor-dependent? To what extent does plasmid architecture influence OMV-mediated transfer?

In this study, to obtain insights into the role of OMVs in the global dissemination of carbapenem resistance, we aimed to examine 2,880 clinical *Acinetobacter* samples collected nationwide from China, of which 10 strains carrying plasmids with the OXA-24-like carbapenemase gene were identified. To elucidate the mechanism of OXA-24-like carbapenemase gene dissemination, we compared the plasmid structures of these ten isolates harboring the *bla*_OXA-24_-like gene and investigated the ability of this gene to be transferred between different *Acinetobacter* strains via OMVs and supernatant.

## MATERIALS AND METHODS

### Bacterial strains and clinical isolates

A total of 2,880 consecutive clinical isolates of *Acinetobacter* spp. were collected from 67 hospitals across 23 provinces in China between January 2009 and December 2010 (with a maximum of 50 isolates per hospital) (11). From this nationwide screening, 10 *Acinetobacter* isolates carrying plasmids with *bla*_OXA-24_-like carbapenemase genes were identified. The isolates included five *A. baumannii* (A1283, A1343, A1429, A2485, and A2503), four *A. pittii* (A352, A2584, A2587, and A2949), and one *A. baylyi* (A2702), all isolated from sputum samples.

Carbapenem-susceptible *Acinetobacter* strains used as recipients were obtained from international culture collections, including the American Type Culture Collection (ATCC), the German Collection of Microorganisms and Cell Cultures, and the Belgian Coordinated Collections of Microorganisms (BCCM/LMG). A carbapenem-resistant *A. baumannii* strain ZJ06, carrying chromosomal *bla*_OXA-23_, was also included (31).

### Plasmid sequencing, typing, and bioinformatics analysis

Plasmids were extracted using a QIAGEN Plasmid Midi Kit and sequenced on an Illumina HiSeq 2000 platform (2 × 100 bp paired end). Reads were assembled using Velvet (v1.1), and remaining gaps were closed by PCR amplification, followed by Sanger sequencing. Primers used for PCR amplification are listed in Table S1. PCR reactions were performed using Vazyme 2× Taq Plus Master Mix (Dye Plus, P212-01), which contains Taq Plus DNA polymerase, dNTPs, and an optimized reaction buffer system. PCR cycling conditions were as follows: initial denaturation at 95°C for 5 min; followed by 35 cycles of 95°C for 30 s, 55°C for 30 s, and extension at 72°C for 45 s; with a final extension at 72°C for 10 min. Nanopore sequencing using a MinION sequencer (Oxford Nanopore Technologies, Oxford, UK) was performed for the genomic DNA of the A2485 strain. Complete sequences were annotated with the RAST server (http://rast.nmpdr.org/rast.cgi), and predicted proteins were verified by BLASTP against the NCBI database. BLASTN was used for homology searches, and comparative plasmid maps were generated with EasyFig.

Plasmids were typed using the Acinetobacter Plasmid Typing scheme (https://github.com/MehradHamidian/AcinetobacterPlasmidTyping) (32, 33). Briefly, *rep* genes were extracted and assigned to Rep_3 replicon types based on greater than 95% nucleotide sequence identity. Plasmids sharing the same or highly similar replication modules were further grouped into the same replicon type.

The identification of p*dif* sites was carried out using the p*dif* tool available at http://bacant.net/pdif/. For the annotation of antimicrobial resistance genes, integrons, and transposable elements, the BacAnt webserver was used (http://bacant.net/BacAnt/) (34). Plasmid visualization and construction were performed using Proksee (v1.6.1) and EasyFig (v2.16.0) (35). The functional annotation of OrfX was conducted using the InterPro database (https://www.ebi.ac.uk/interpro/).

### OMV isolation

OMVs were purified from bacterial cultures by ultracentrifugation. A single fresh colony was inoculated into 10 mL of Mueller-Hinton (MH) broth supplemented with 10 μg/mL meropenem and incubated overnight at 37°C with shaking (180 rpm). A total of 5 mL of this culture was used to inoculate 500 mL of MH broth under the same conditions and grown to an OD₆₀₀ of 1.0. Bacterial cells were pelleted (14,000 × *g*, 10 min), and the supernatant was collected and filtered through a 0.22-μm sterile membrane (Millipore, USA) to remove residual bacteria and debris. The filtrate was subjected to ultracentrifugation (200,000 × *g*, 90 min, 4°C, 70Ti rotor, Beckman). The resulting OMV pellet was resuspended in PBS (pH 7.4), passed through a 0.22 μm sterile filter, and a small aliquot was plated on MH agar to verify sterility. OMVs were stored at –80°C until use.

### Total OMV protein content

Total OMV protein content was quantified using a BCA assay (Thermo Scientific, A65453). Standard protein solutions (0–0.5 mg/mL) were prepared in 96-well plates with a final volume of 20 μL per well. OMV samples were diluted to obtain a final volume of 20 μL and examined. BCA working solution (200 μL) was added to each well, and the plate was incubated at 37°C for 20–30 min. Absorbance was measured at 562 nm using a microplate reader. Protein concentrations were determined from the standard curve, adjusted for sample volume. Total OMV-associated protein quantification was performed in three biological replicates.

### Transmission electron microscopy

Bacterial cells and OMVs were examined by transmission electron microscopy (TEM). A single colony was grown overnight in MH broth (with 10 μg/mL meropenem for resistant strains), harvested, and fixed in 2.5% glutaraldehyde at 4°C overnight. Samples were rinsed with phosphate buffer, post-fixed with 1% osmium tetroxide, dehydrated in a graded ethanol series, and treated with acetone. Samples were embedded in EPON 812 resin, polymerized sequentially at 37°C, 45°C, and 60°C, and sectioned. Ultrathin sections (70 nm) were cut, double stained with 2% uranyl acetate and lead citrate, and imaged using a 100 kV TEM (TECNAI 10, Philips) with a Gatan CCD camera (830.P24W44). For direct OMV observation, 5 μL of OMV suspension was applied to a glow-discharged carbon-coated copper grid for 1 min, stained with 1% uranyl formate, and imaged under identical TEM conditions.

### OMV-mediated transformation assay

OMV-mediated transformation was performed as described by Yaron et al. (36) with minor modifications. *A. baumannii* ATCC 17978 was grown in MH broth to mid-log phase (OD₆₀₀ ≈ 0.4), centrifuged, and resuspended in fresh MH broth to the same OD. A total of 20 μg of OMVs (quantified by BCA assay) was added to 60 μL of recipient cells. The mixture was incubated statically at 37°C for 2 h, followed by shaking at 200 rpm for another 2 h. Cultures were supplemented with 10 mL fresh MH broth and incubated overnight. Cells were harvested, serially diluted, and plated on MH agar with or without 10 μg/mL meropenem. Transformation frequency was calculated as the ratio of transformants to total CFU. Controls included free plasmid DNA from A2485 (equivalent to the DNA amount in OMVs), OMVs pre-treated with 0.125% Triton X-100, and recipient cells without OMVs. All experiments were performed in duplicate.

### Direct transformation assay via culture supernatant

To evaluate resistance gene transfer without OMV purification, culture supernatant from donor *A. baumannii* A2485 (grown in MH broth containing 10 μg/mL meropenem) was collected, centrifuged, and filtered (0.22 μm) to remove residual cells; sterility was verified by plating. Recipient strain ATCC 17978 was cultured to mid-log phase (OD₆₀₀ ≈ 0.4), adjusted to the same OD, and mixed (200 μL each) with sterile supernatant. Mixtures were incubated sequentially at 37°C under static and shaking conditions, then supplemented with fresh MH broth and cultured overnight. Cells were harvested, serially diluted, and plated on MH agar with or without 10 μg/mL meropenem to determine total CFU and transformants. Transformation frequency was calculated as transformants/total CFU. Controls included supernatant pretreated with 0.125% Triton X-100 and recipient cells without supernatant. All assays were performed in duplicate.

### PCR amplification

Presence of *bla*_OXA-72_ in transformant colonies was confirmed by PCR. Primers used were Forward: 5′-GGTTAGTTGGCCCCCTTAAA-3′ and Reverse: 5′-AGTTGAGCGAAAAGGGGATT-3′. DNA templates were prepared by boiling bacterial suspensions. PCRs were performed using Vazyme 2× Taq Plus Master Mix (Dye Plus, P212-01). Each 25 μL reaction contained 12 μL 2× Master Mix, 0.5 μL forward primer, 0.5 μL reverse primer, 1 μL DNA template, and 11 μL nuclease-free water. PCR cycling conditions were as follows: initial denaturation at 98°C for 1 min; followed by 35 cycles of 98°C for 10 s, 50°C for 30 s, and 72°C for 30 s; with a final extension at 72°C for 10 min. Positive PCR products were subjected to Sanger sequencing, which was performed by Beijing Tsingke Biotech Co., Ltd.

### Pulsed-field gel electrophoresis

Transformant genetic backgrounds were verified by pulsed-field gel electrophoresis (PFGE). Cultures were grown for 8 h, adjusted to 4 McFarland units, and embedded in low-melting-point agarose plugs. Plugs were lysed, digested with a restriction enzyme, and subjected to electrophoresis on a CHEF-DR III system (Bio-Rad) under optimized conditions. Banding patterns were compared to confirm transformant identity with the recipient strain.

### Antimicrobial susceptibility testing

Antimicrobial susceptibility was assessed using broth microdilution, interpreted according to CLSI 2024 guidelines (37). Cefoperazone breakpoints were applied for cefoperazone/sulbactam. Colonies grown overnight on MH agar (Oxoid) at 37°C were suspended in sterile 0.85% NaCl. The turbidity was adjusted to a 0.5 McFarland standard using a DensiCHEK Plus instrument (bioMérieux, France). The suspension was then diluted 1:100 in cation-adjusted Mueller-Hinton broth (CAMHB) and inoculated into 96-well plates (50 μL suspension + 50 µL serially diluted antibiotics in CAMHB). MICs were defined as the lowest concentration preventing visible growth after 20 h at 35°C. MICs were determined in triplicate.

### Preparation of competent cells and electroporation

Competent *Acinetobacter* cells were prepared by culturing to mid-log phase (OD₆₀₀ ≈ 0.5–0.6). Cells were chilled on ice for 15 min and harvested by centrifugation (3,000 × *g*, 10 min, 4°C). The pellet was washed once with ice-cold sterile water and three times with 10% glycerol. Between washes, cells were pelleted by centrifugation (3,000 × *g*, 10 min, 4°C). Finally, cells were resuspended in 10% glycerol and used immediately or stored at −80°C. For electroporation, approximately 400 ng of plasmid DNA was mixed with 100 μL of competent cells. Plasmid DNA was mixed with competent cells in pre-chilled electroporation cuvettes, incubated on ice, and electroporated (Bio-Rad Gene Pulser; 2.5 kV, 200 Ω, 25 μF). Cells recovered in MH broth at 37°C for 1 h were plated on MH agar with 10 μg/mL meropenem to select transformants.

### Determination of DNA and proteins in OMVs

Determination of DNA and proteins in OMVs was performed as described by Rumbo et al. with minor modifications (29). Purified OMV samples (100 µL) were first normalized to equal protein concentrations based on BCA quantification (e.g., adjusted to 0.5 mg/mL). To remove extravesicular DNA, DNase I was added to a final concentration of 1 ng/mL, followed by incubation at 37°C for 1 h under static conditions and an additional 2 h at 150 rpm to digest free DNA associated with the vesicle surface or present in the suspension. The reaction was subsequently terminated by heat inactivation at 80°C for 10 min to prevent degradation of intravesicular DNA released in later steps.

For vesicle lysis, Triton X-100 was added to a final concentration of 0.125%, and samples were incubated at 37°C for 30 min to disrupt the lipid bilayer and release encapsulated DNA. The released DNA was then purified using the Pure PCR product purification kit (Zymo Research), including washing steps to remove lipid and protein contaminants. Detection of the *bla*_OXA-72_ gene was performed by conventional PCR.

### Statistical analysis

Analyses were performed with SPSS (v17.0). Categorical variables were compared using the χ² test, and continuous variables with Student’s *t*-test. *P* < 0.05 was considered statistically significant. Graphs were generated using GraphPad Prism (v7.04).

## RESULTS

### Rare occurrence of *bla*_OXA-24_-like plasmids in *Acinetobacter* and insights into their architecture

Nationwide screening of 2,880 *Acinetobacter* clinical isolates identified 10 strains carrying *bla*_OXA-24/72_ plasmids, comprising five *A. baumannii*, four *A. pittii*, and one *A*. *baylyi*, all recovered from sputum samples. Sequencing revealed nine distinct plasmids, eight carrying *bla*_OXA-72_ and one carrying *bla*_OXA-24_. Plasmid sizes ranged from 7,852 bp (*p*A1429) to 20,526 bp (*p*A1283), with GC contents of 31.99%–35.46% and 9–24 predicted ORFs. Replicon typing resolved four groups: group A (R3-T24/R3-T13 and R3-T13), group B (R3-T5/R3-T8, R3-T2/R3-T8, and R3-T8; the largest group), group C (R3-T97), and group D (R3-T18) (Table 1).

**TABLE 1:** Characteristics and conservation of major functional modules in *bla*_OXA-24/72_-carrying Rep_3 plasmids of *Acinetobacter* strains

Strain	Species	Province	Plasmid name	Size (bp)	Rep type	Group	Type II TA module	Mobilization module	C module backbone	p*dif* sites (no.)^*a*^	Iterons (no.)	*orfX* (bp)	ORFs (no.)
A352	*A. pittii*	Liaoning	*p*A352	13,790	R3-T24, R3-T13	A	VapBC (←)	Absent	Yes	6	4	480/525	19
A1283	*A. baumannii*	Hainan	*p*A1283	20,526	R3-T5, R3-T8	B	VapBC; HigBA (*higBA2*) (→)	*mobA*	Yes	6	4	558/546	24
A1343	*A. baumannii*	Hainan	*p*A1343	8,539	R3-T13	A	VapBC (→)	Absent	Yes	4	5	510	12
A1429	*A. baumannii*	Guangdong	*p*A1429	7,852	R3-T18	D	Xre-like/RelE-like (←)	*mobS*; *mob*L-like	No	2	4	None	9
A2485	*A. baumannii*	Shanxi	*p*A2485	15,405	R3-T2, R3-T8	B	VapBC (→)	*mobA*	Yes	6	4	558/576	21
A2503	*A. baumannii*	Shanxi	*p*A2503	15,405	R3-T2, R3-T8	B	VapBC (→)	*mobA*	Yes	6	4	558/576	20
A2584	*A. pittii*	Jiangxi	*p*A2584	8,166	R3-T8	B	None detected	*mobA*	Yes	4	4	558	10
A2587	*A. pittii*	Jiangxi	*p*A2584^*b*^	8,166	R3-T8	B	None detected	*mobA*	Yes	4	4	558	10
A2702	*A. baylyi*	Jiangxi	*p*A2702	10,750	R3-T8	B	VapBC (←); YefM/YoeB (→)	*mobA*	Yes	4	4	459	14
A2949	*A. pittii*	Hubei	*p*A2949	10,886	R3-T97	C	HigBA (*higBA2*) (→)	Absent	Yes	6	4	543	17

^*a*^ p*dif* sites (no.) refer to the number of XerC/XerD recombination sites (C/D and D/C) identified in each plasmid.

^*b*^ The plasmid *p*A2587 was identical in sequence to *p*A2584.

Eight of the nine non-redundant plasmids, corresponding to nine of the ten plasmids identified, conformed to the recently defined Rep_3/OrfX C-module backbone structure, comprising iterons–*repA–orfX* bounded by D/C and C/D p*dif* sites, with accessory *dif* modules carrying resistance and stability genes (23). The sole exception, *p*A1429 (Group D), lacked both the C-module backbone and *orfX*, and uniquely carried a *mobS*/*mobL*-like mobilization module. Among the remaining nine, *mobA* was detected in six; three plasmids (*p*A352, *p*A1343, *p*A2949) lacked any identifiable mobilization locus.

**Fig 1:**
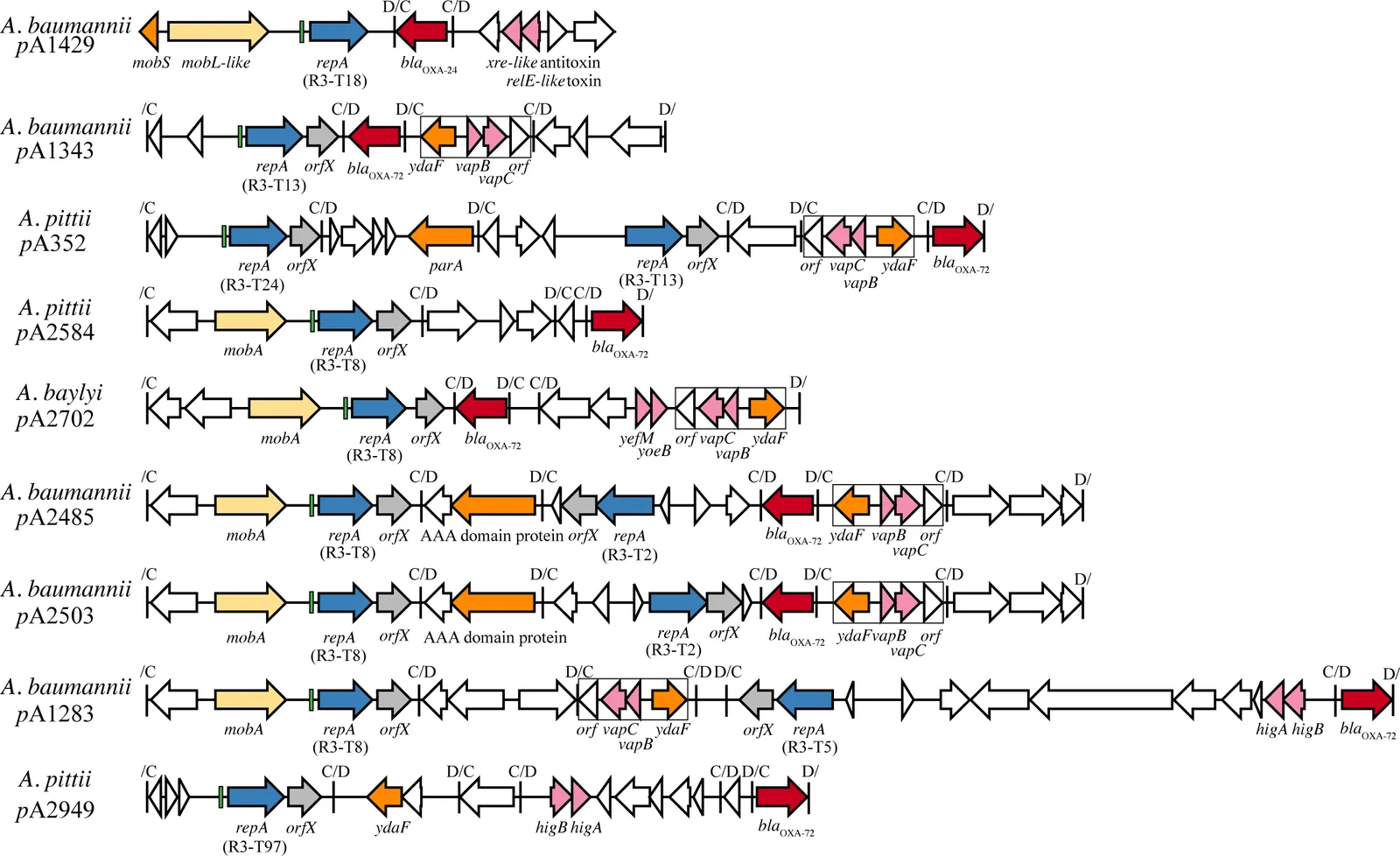
Conserved modular architecture of *bla*_OXA-24_-like plasmids across *Acinetobacter* species. Schematic representation of the modular architecture of Rep_3 family plasmids harboring *bla*_OXA-24_ or *bla*_OXA-72_ identified from a nationwide survey of 2,880 clinical *Acinetobacter* isolates. Arrows indicate the extent and direction of coding sequences, with gene annotations shown below each feature. The Rep_3-type replication initiation gene *repA* is shown in light blue, the downstream conserved open reading frame *orfX* in gray, mobilization-associated genes in yellow, carbapenemase genes (*bla*_OXA-24/72_) in red, putative toxin–antitoxin modules in pink, and hypothetical open reading frames in white. Iteron repeats upstream of *repA* are indicated by a green box. The boxed region represents an approximately 2 kb conserved segment associated with the VapBC toxin–antitoxin system, comprising the gene arrangement *orf–vapC2–vapB2–ydaF*. XerC/XerD recombination sites (*pdif*) are shown as vertical bars with orientation labels (C/D or D/C), following correction of site directionality across all plasmids.

All ten plasmids, representing nine non-redundant plasmid sequences, carried a *blaOXA*-p*dif* resistance module (2–6 p*dif* sites total). Type II toxin–antitoxin (TA) systems were variably distributed among the plasmids. VapBC was the most prevalent system, being detected in six plasmids. In addition to VapBC, *p*A1283 co-harbored HigBA (*higBA2*), whereas *p*A2702 co-harbored YefM/YoeB. HigBA (*higBA2*) alone was detected in *p*A2949. An Xre-like/RelE-like system was exclusively identified in *p*A1429, while two plasmids, *p*A2584 and *p*A2587, lacked any detectable TA locus. Collectively, these plasmids share a conserved C-module backbone and a universally distributed *blaOXA*-p*dif* resistance module, superimposed on a mosaic accessory architecture spanning four replicon groups (Table 1, Fig. 1; Fig. S1).

Comparative genomic analysis identified a cointegrate structure in *p*A352, comprising two distinct backbone regions: one with near-complete sequence identity to *A. baumannii* plasmid *p*AbIHIT32296 (KY704308.1), and another with high similarity to *p*SIMBA113_2 (CP162302.1). Conserved backbone genes (*repA*, *repM*, *parA*) and TA system genes (*vapC*/*vapB*) were identified within the respective regions (Fig. S2).

### Genetic characterization of plasmid *p*A2485 and the chromosomal resistome of strain A2485

Sequencing of plasmid *p*A2485 (15,405 bp) confirmed the presence of *bla*_OXA-72_ within a Rep_3/OrfX C-module framework, while *bla*_OXA-23_ was not detected, correcting a previous preliminary report based on PCR (11). The plasmid contains two separate backbone regions. The first backbone, corresponding to R3-T8, comprises *mobA*, *repA*, and *orfX*, while the second backbone, corresponding to R3-T2, comprises an AAA-domain protein and a replication-associated region. A type II VapBC toxin–antitoxin module is encoded within a 1,781-bp region (*orf–vapC2–vapB2–ydaF*). These modules are organized as discrete units bounded by p*dif* sites, with three XerC/XerD and three XerD/XerC boundary pairs, consistent with p*dif*-mediated modular organization (Fig. 1).

Chromosomal analysis of strain A2485 showed that it belonged to ST2, consistent with a GC2 background. Two intrinsic β-lactamase loci were identified: *bla_OXA-66_*, an OXA-51-like carbapenemase gene, and *bla_ADC-30_*, an AmpC-type cephalosporinase gene. The upstream IS*Aba1* insertion associated with *bla_ADC-30_* is known to promote *ampC* overexpression and confer resistance to cephalosporins. Several acquired resistance genes were located within known chromosomal resistance islands, including an AbGRI1-1-like region carrying *tet(B*), *strA* [*aph*(3″)-*Ib*], and *strB* [*aph*(6)-*Id*], and an AbGRI3-2-like region carrying *armA*, *msr*(E), *mph*(E), *catB8*, *sul1*, *aadA1*, and *aphA1* [*aph*(3′)-*Ia*]. No AbGRI2 island was detected. Other chromosomally encoded resistance-associated determinants included *amvA*, *abaF*, and *adeC* (Fig. S3, Tables S2 to S4).

### Increased total OMV-associated protein content in carbapenem-resistant *A. baumannii* carrying the *bla*_OXA-72_ plasmid

To investigate the reasons underlying the limited dissemination of *bla*_OXA-72_ plasmids in *Acinetobacter* strains and whether OMVs could mediate the spread of this resistant plasmid, we selected A2485 (carrying *bla*_OXA-72_ on a plasmid) as the representative strain and ZJ06 (carrying *bla*_OXA-23_ on the chromosome) as the control.

**Fig 2:**
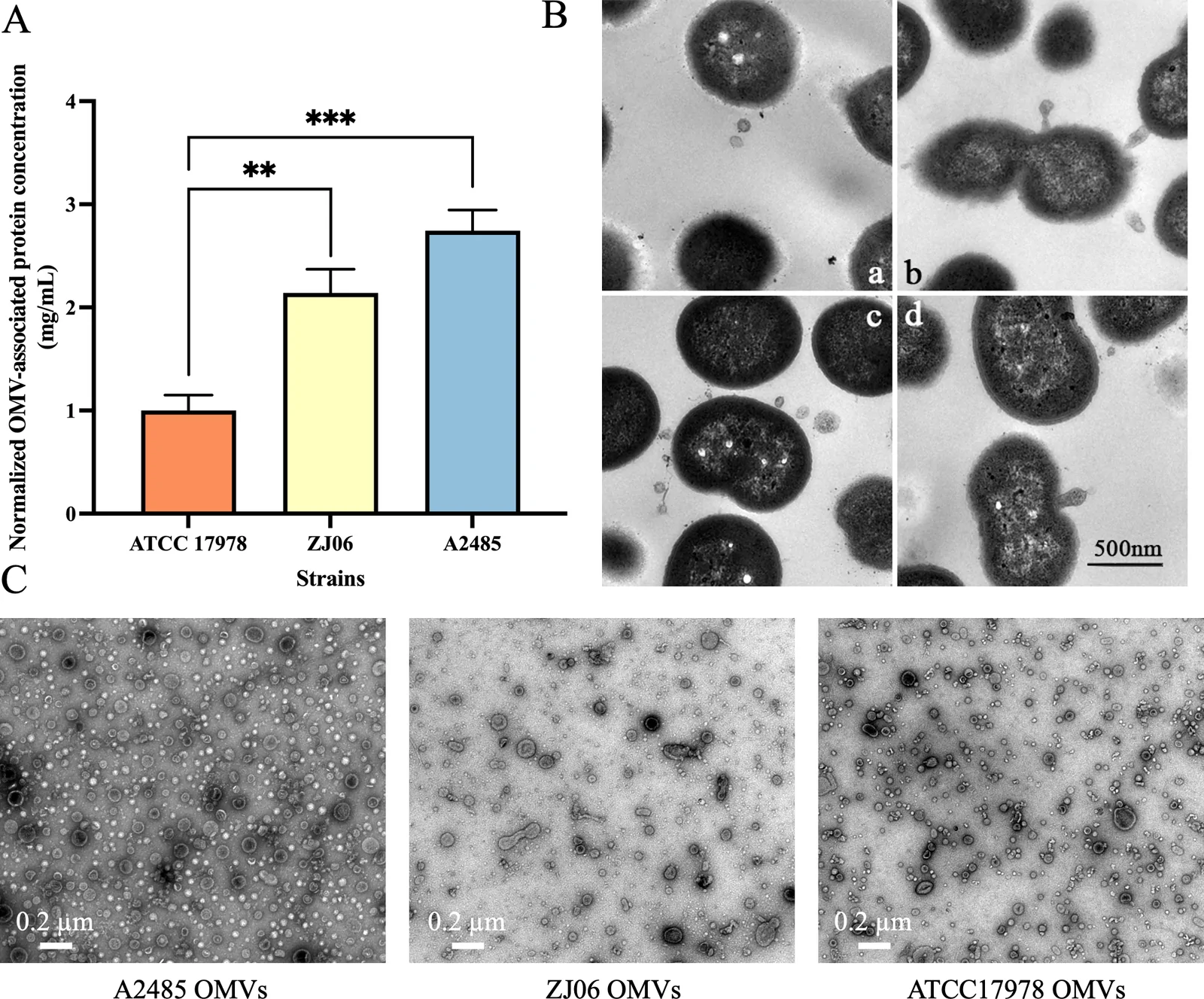
Characterization of OMV biogenesis and morphology in *A. baumannii* strains. (**A**) Quantification of total OMV protein content (determined by BCA assay) in clinical strains A2485 and ZJ06 compared with laboratory strain ATCC 17978. Statistical significance was assessed using unpaired two-tailed Student’s *t*-test. *P* < 0.01 (**), *P* < 0.001 (***). (**B**) TEM images of OMV release. (a) A2485: extracellular spherical OMVs. (b) ATCC 17978: OMVs budding from the outer membrane. (c) ZJ06: multiple extracellular OMVs of varying sizes. (d) ZJ06: vesicle connected to the bacterial division septum via a tubular structure. (**C**) TEM images of purified OMVs. All images were captured at a magnification of 17K.

Total OMV protein levels, quantified by BCA assay and normalized to the mean value of ATCC 17978, were compared across strains to account for inter-replicate variability. A2485 exhibited significantly higher total OMV-associated protein content than ATCC 17978 (2.74-fold ± 0.20-fold, *P* < 0.001), while ZJ06 showed a moderate increase (2.14-fold ± 0.23-fold, *P* = 0.002) (Fig. 2A).

TEM revealed vesicle budding from the outer membrane, particularly at division septa, with vesicle diameters of 20–300 nm (Fig. 2B). Isolated OMVs appeared intact and abundant in all strains. A2485-derived OMVs were uniform, spherical, and averaged ~60 nm in diameter, whereas those from ATCC 17978 were smaller (~40 nm) (Fig. 2C). No bacterial contamination was detected in OMV preparations, confirming isolation purity.

### OMV-mediated transfer of *bla*_OXA-72_ plasmid to *A. baumannii*

**Fig 3:**
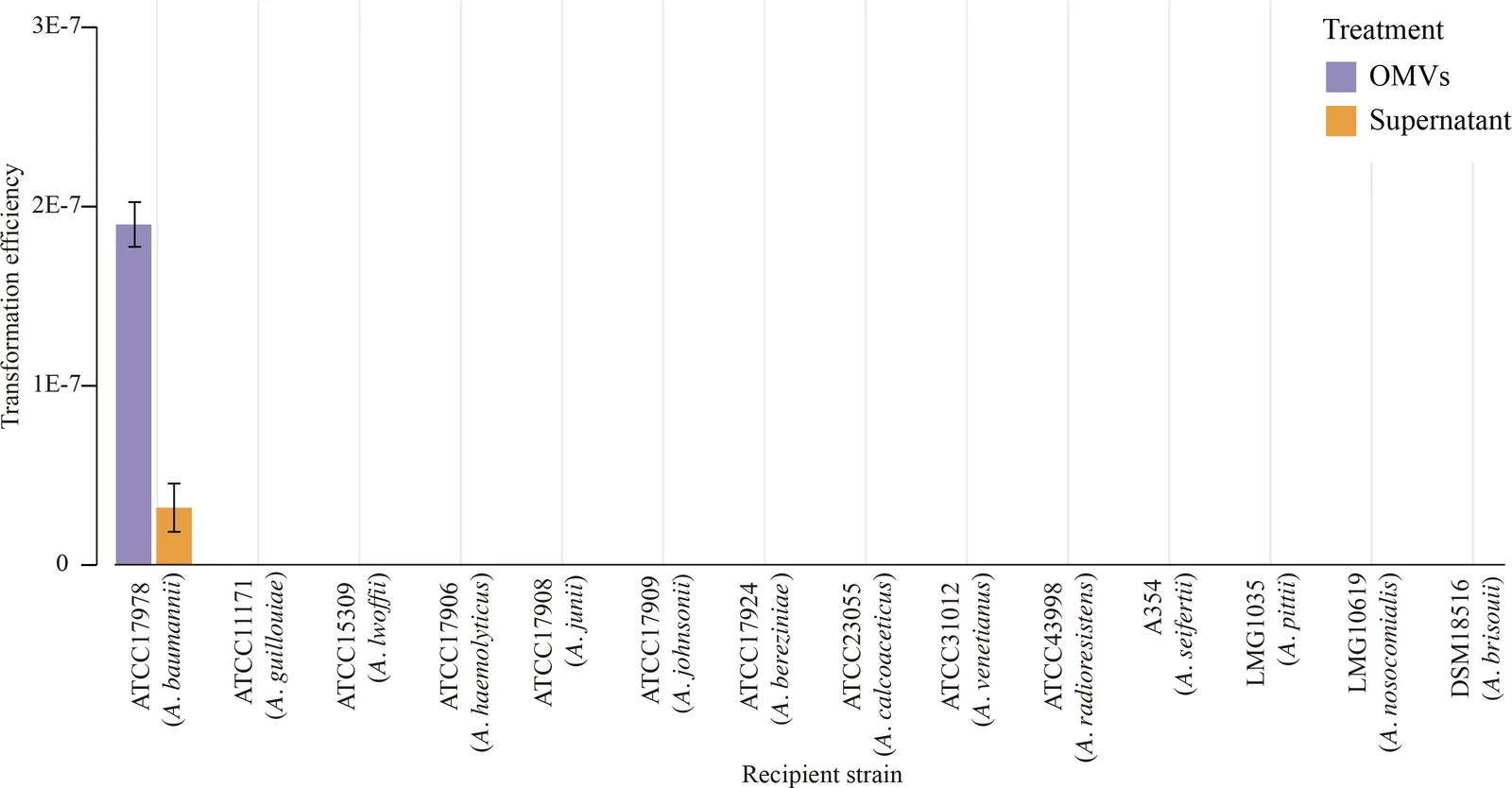
Strain-specific OMV-mediated plasmid transfer in *A. baumannii*. OMVs and culture supernatant derived from donor strain A2485 were assessed for their ability to transfer *bla*_OXA-72_ to different *Acinetobacter* recipient strains. Efficient transformation was observed only in *A. baumannii* ATCC 17978, whereas no detectable transfer occurred in 13 non-*baumannii Acinetobacter* species. Transformation frequencies in ATCC 17978 were 1.9 × 10⁻⁷ transformants per CFU for OMVs and 3.2 × 10⁻⁸ transformants per CFU for culture supernatant. Purple bars indicate OMVs, and orange bars indicate culture supernatant. Error bars represent mean ± SD.

OMVs from A2485 successfully transferred a *bla*_OXA-72_ plasmid to ATCC 17978, yielding transformants (17978V) at a frequency of 1.9 × 10⁻⁷ transformants per CFU, confirmed by PCR and PFGE. We also found that the supernatant from A2485 was able to directly transfer the *bla*_OXA-72_ plasmid to ATCC 17978 (designated as 17978S after transformation) at a frequency of 3.2 × 10⁻⁸ transformants per CFU, slightly lower than the transformation frequency mediated by OMVs. In comparison, neither OMVs nor the supernatant from ZJ06 were able to mediate the transfer of the *bla*_OXA-23_ gene (Fig. 3, Table 2; Fig. S4, Table S5).

**TABLE 2:** Transformation frequency of *A. baumannii* ATCC 17978 mediated by different donor types

Donor type	Transformation frequency (transformants per CFU)^*d*^
A2485 OMVs	1.900 × 10⁻⁷ ± 1.250 × 10⁻⁸
A2485 OMVs + Triton X-100^*a*^	0
ZJ06 OMVs	0
ZJ06 OMVs + Triton X-100^*a*^	0
17978 OMVs	0
17978 OMVs + Triton X-100^*a*^	0
A2485 supernatant	3.200 × 10⁻⁸ ± 1.348 × 10⁻⁸
A2485 supernatant + Triton X-100^*a*^	0
A2485 plasmid^*b*^	0
MH broth^*c*^	0

^*a*^ Pretreated with 0.125% Triton X-100 at 37°C for 30 min.

^*b*^ Plasmid DNA extracted from A2485.

^*c*^ Mueller-Hinton broth used as a negative control.

^*d*^ Transformation frequency represents the mean ± SD of two independent experiments.

Antimicrobial susceptibility testing showed that transformants (17978V and 17978S) displayed significantly increased resistance to meropenem, ceftazidime, cefoperazone/sulbactam, and piperacillin/tazobactam compared to parental ATCC 17978 (Table 3).

**TABLE 3:** MICs of β-lactam antibiotics against donor strains, recipient strain, and *bla*_OXA-72_ transformants generated by OMV- and supernatant-mediated transfer

Strains^*a*^	MIC^*b*^ (µg/mL)			
	MEM	CAZ	SCF	TZP
17978V	>128	>128	64	16
17978S	>128	>128	64	32
A2485	>128	>128	128	128
17978	0.25	16	4	16

^*a*^ 17978V: ATCC 17978 transformant obtained by A2485 OMVs; 17978S: ATCC 17978 transformant obtained by A2485 supernatant.

^*b*^ MEM, meropenem; CAZ, ceftazidime; SCF, cefoperazone/sulbactam (2:1); TZP, piperacillin/tazobactam.

### OMV-mediated transfer of *bla*_OXA-72_ plasmids fails in non-*A*. *baumannii* strains

To investigate whether OMVs from A2485 can transfer the *bla*_OXA-72_ plasmid to non-*A*. *baumannii* (nAB) strains, we selected 13 nAB strains as recipients. None of the 13 nAB strains were able to acquire the *bla*_OXA-72_ plasmid from A2485-derived OMVs, including strains such as *A. pittii* LMG1035, *A. guillouiae* ATCC 11171, *A. haemolyticus* ATCC 17906, and *A. nosocomialis* LMG10619. Similarly, we attempted to use the supernatant from A2485 to transform these 13 nAB strains, but the supernatant was also ineffective in transferring the *bla*_OXA-72_ plasmid to nAB strains (Fig. 3).

To rule out the possibility that the inability of the *bla*_OXA-72_ plasmid to transfer to nAB strains was due to plasmid-related factors, we extracted the pA2485 plasmid from A2485 and performed electroporation into the 13 nAB strains. The results showed that pA2485 could successfully transfer to seven of the nAB strains (*A. guillouiae* ATCC 11171, *A. junii* ATCC 17908, *A. venetianus* 31012, *A. seifertii* A354, *A. pittii* LMG1035, *A. nosocomialis* LMG10619, and *A. brisouii* DSM18516), indicating that these nAB strains are capable of accepting the *bla*_OXA-72_ plasmid but cannot receive it through OMVs or supernatants.

### OMV-mediated plasmid transfer is restricted to the original donor strain and does not extend across species

To determine whether plasmid-acquiring recipients could subsequently act as OMV donors, we first generated two *A. baumannii* ATCC 17978 derivatives carrying pA2485: strain 17978E, obtained by electroporation, and strain 17978V, which had acquired the plasmid via OMV-mediated transformation. Culture supernatants from both strains were then used as potential donors in transformation assays with a fresh ATCC 17978 recipient. No transformants were recovered from either 17978E or 17978V, whereas the original clinical strain A2485 consistently yielded successful transfer under identical conditions (Table 4). These results indicate that plasmid transfer is not a general property conferred by plasmid possession alone, but is instead specific to the original donor strain A2485, suggesting that strain-intrinsic factors, such as OMV surface composition, production efficiency, or plasmid mobilization machinery, are required for competent OMV-mediated transfer.

**TABLE 4:** Direct transformation assay of *bla*_OXA-72_ OMVs-transformed and electroporated culture supernatants into *A. baumannii* and non-*A*. *baumannii* strains^*d*^

Donor supernatant^*a*^	Recipient strain	Transformation result
A2485^*b*^	ATCC 17978(*A. baumannii*)	+
17978E		-
17978V		-
11171E		-
17908E		-
31012E		-
A354E		-
LMG1035E		-
LMG10619E		-
DSM18516E		-
11171E	ATCC 11171 (*A. guillouiae*)	-
17908E	ATCC 17908 (*A. junii*)	-
31012E	ATCC 31012 (*A. venetianus*)	-
A354E	A354 (*A. seifertii*)	-
LMG1035E	LMG1035 (*A. pittii*)	-
LMG10619E	LMG10619 (*A. nosocomialis*)	-
DSM18516E	DSM18516 (*A. brisouii*)	-
MH Broth^*c*^	ATCC17978 (*A. baumannii*)	-

^*a*^ Strains with “E” are* p*A2485 electrotransformed with *bla*_OXA-72_, while those with “V” are *p*A2485 OMVs-transformed with *bla*_OXA-72_.

^*b*^ Supernatant of A2485 used as a positive control.

^*c*^ Mueller-Hinton broth used as a negative control.

^*d*^ The gray-shaded area indicates assays in which ATCC 17978 (*A. baumannii*) was used as the recipient strain.

To assess whether this donor specificity extends across species boundaries, we electroporated *p*A2485 into seven nAB strains and prepared culture supernatants for use as donors in transfer assays targeting both ATCC 17978 and the corresponding nAB recipients. No successful transfer was observed in any combination tested, demonstrating that nAB strains fail to act as effective OMV donors regardless of recipient species identity (Table 4).

### OMV biogenesis and plasmid packaging occur across strains but transfer competence is governed by post-packaging, donor-specific barriers

**Fig 4:**
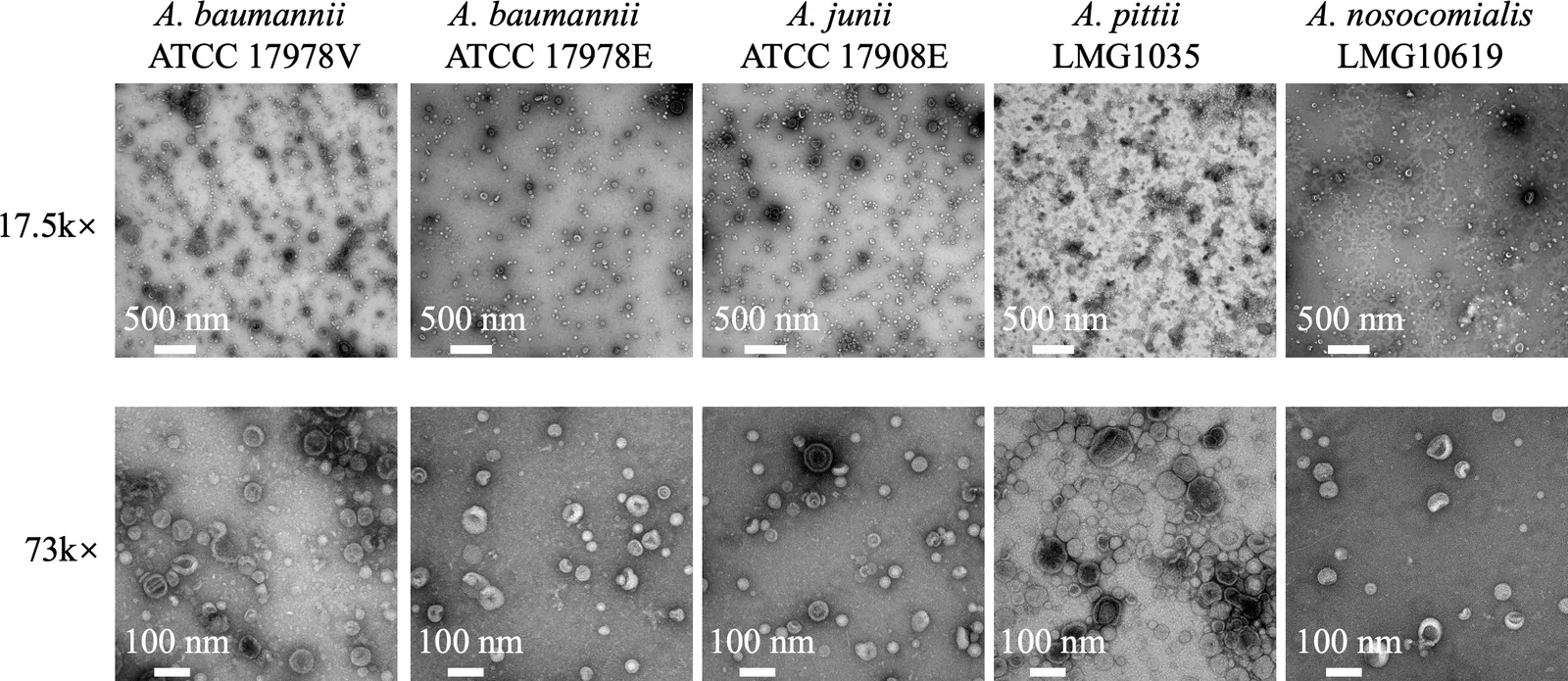
TEM analysis of OMVs from five *Acinetobacter* strains. TEM images of negatively stained, purified OMVs isolated from *A. baumannii* ATCC 17978V, *A. baumannii* ATCC 17978E, *A. junii* ATCC 17908E, *A. pittii* LMG1035E, and *A. nosocomialis* LMG10619E. Upper panels (17.5k×) provide an overview of OMV distribution and density, with scale bars = 500 nm. Lower panels (73k×) show high-magnification views revealing OMV ultrastructure, including the characteristic bilayer membrane architecture and heterogeneity in vesicle size, with scale bars = 100 nm. OMVs from all five strains display a spherical morphology with an intact double-membrane boundary.

To determine whether the absence of transfer from 17978E, 17978V, and nAB strains reflected a defect in OMV biogenesis or DNA packaging, we systematically examined both processes. Transmission electron microscopy confirmed that all five strains (ATCC 17978E, ATCC 17978V, *A. junii* ATCC 17908E, *A. pittii* LMG1035E, and *A. nosocomialis* LMG10619E) produced morphologically intact OMVs, ruling out a vesicle biogenesis defect (Fig. 4). DNase I protection assays, followed by Triton X-100 lysis and intravesicular DNA extraction, confirmed the presence of *p*A2485 within OMVs from all five strains, establishing that plasmid packaging occurs in both *A. baumannii* and nAB strains (Fig. S5).

Quantification of intra-vesicular DNA content revealed host-dependent differences in packaging efficiency. *A. junii* 17908E and *A. pittii* LMG1035E packaged substantially less DNA per unit of OMV protein (~4.5–4.8 ng/μg) compared to *A. baumannii* strains (~13.6–28.6 ng/μg). *A. nosocomialis* LMG10619E showed an intermediate level (~11.7 ng/µg) yet still failed to mediate transfer. Critically, *A. baumannii* strains 17978E and 17978V packaged comparable or greater amounts of DNA relative to the original donor A2485 (28.62, 23.98, and 13.62 ng/μg, respectively), yet completely failed to transfer the plasmid (Table 5). These findings demonstrate that packaging efficiency alone is insufficient to confer donor competence, and implicate post-packaging barriers as the primary constraint on transfer in these strains.

**TABLE 5:** Intra-vesicular DNA-to-protein ratios of OMVs isolated from *Acinetobacter* strains carrying *p*A2485

Source^*a*^	Species	Plasmid	DNA/protein (ng/μg)
A2485	*A. baumannii*	*p*A2485	13.62
ATCC 17978E	*A. baumannii*		28.62
ATCC 17978V	*A. baumannii*		23.98
17908E	*A. junii*		4.80
LMG1035E	*A. pittii*		4.50
LMG10619E	*A. nosocomialis*		11.73

^*a*^ Strains with “E” are *p*A2485 electrotransformed with *bla*_OXA-72_, while that with “V” are *p*A2485 OMVs-transformed with *bla*_OXA-72_.

## DISCUSSION

The global rise of carbapenem-resistant *Acinetobacter* spp. underscores the need to clarify the mechanisms driving their dissemination (1). OXA-24-like carbapenemases are widespread in Europe, whereas only sporadic reports exist in China, and the reasons for their limited spread remain unclear (15). Nationwide screening of 2,880 clinical *Acinetobacter* isolates identified only 10 strains carrying *bla*_OXA-24/72_ plasmids across three species, with *bla*_OXA-72_ predominating. The absence of clonal expansion, with the exception of A2584 and A2587, which belong to the same clone, suggests that clonal dissemination contributes minimally to the spread of *bla*_OXA-24_-like genes in China.

The nine plasmids conforming to the Rep_3/OrfX C-module backbone share a conserved structural framework (iterons–*repA–orfX* bounded by D/C and C/D p*dif* sites), consistent with previously described replicon architectures in which the backbone serves as a scaffold for resistance gene acquisition (23). The sole exception, *p*A1429, lacked both the C-module backbone and *orfX* and uniquely carried a *mobS/mobL*-like mobilization module, suggesting it represents a structurally divergent lineage within this plasmid family. The universal presence of *blaOXA*-p*dif* modules across all 10 plasmids highlights the central role of p*dif*-mediated recombination in the horizontal mobilization of carbapenem resistance determinants. XerC/XerD-mediated site-specific recombination at p*dif* sites facilitates intramolecular shuffling and intermolecular plasmid fusions, driving the spread of *bla*_OXA-24_-like genes across diverse plasmid scaffolds (38, 39). The cointegrate structure identified in *p*A352, comprising backbone regions with high similarity to two distinct reference plasmids, further illustrates the plasticity of this recombination system in generating novel plasmid architectures.

Type II TA systems were variably but frequently detected across the plasmid collection. The VapBC system, present in six plasmids, including pA2485, plays a well-established role in plasmid stabilization and is known to co-mobilize with resistance genes, such as *bla*_OXA-72_, potentially preventing plasmid loss under nutrient-limited conditions (40–42). Additional systems, including HigBA, YefM/YoeB, and the Xre-like/RelE-like module, further contribute to plasmid maintenance across different replicon groups (43). Notably, two plasmids, pA2584 and pA2587, lacked any detectable toxin–antitoxin locus, raising the question of whether alternative stability mechanisms operate in these replicons.

OMVs are recognized mediators of HGT, capable of packaging and protecting plasmids and resistance genes (29, 30, 36). In *Enterobacterales*, OMVs have been shown to transfer carbapenemase plasmids, such as *bla*_OXA-232_, across diverse recipients, supporting their designation as a broad-spectrum horizontal gene transfer vehicle (44). Consistent with previous reports, our carbapenem-resistant clinical strains exhibited higher total OMV protein content than the laboratory strain ATCC 17978, suggesting that antibiotic pressure may be associated with increased OMV-associated protein content (45, 46). Functional assays confirmed that OMVs mediate plasmid transfer at efficiencies substantially exceeding those of free DNA uptake, acting as a protective vehicle for plasmid delivery, and detergent treatment abolished transfer entirely, confirming dependence on intact lipid bilayers (29, 30, 36).

Our study confirms OMV-mediated plasmid transfer in *Acinetobacter* but reveals a pattern strikingly distinct from that observed in *Enterobacterales*. The selection of A2485 as the sole donor strain in these experiments was deliberate. Among the nine *bla*_OXA-72_-carrying plasmids identified in our collection, *p*A2485 was selected as a representative of the recurrent Group B plasmid lineage, as it shares a highly conserved backbone structure with *p*A2503. In addition, restricting experiments to a single, fully sequenced plasmid eliminated variables arising from differences in replicon structure, including backbone diversity, accessory cargo, and TA composition, that would otherwise complicate interpretation of transfer outcomes. We acknowledge that this approach does not address the transfer behavior of other plasmids in our collection, which represents a limitation of this study and an important direction for future investigation.

Using the clinical *bla*_OXA-72_-positive strain A2485 as donor, OMVs efficiently transferred *p*A2485 to *A. baumannii* ATCC 17978, conferring resistance to meropenem, ceftazidime, and cefoperazone/sulbactam. In contrast, this process was strictly confined to *A. baumannii* and failed entirely across 13 non-*A*. *baumannii* strains, demonstrating a pronounced species barrier (44, 47). To dissect the mechanistic basis of this barrier, we examined OMV biogenesis and plasmid packaging across non-donor strains. Transmission electron microscopy confirmed that all tested strains produced morphologically intact vesicles, and DNase I protection assays demonstrated active plasmid encapsulation in both *A. baumannii* derivatives and nAB strains alike. Quantification of intra-vesicular DNA revealed that strains 17978E and 17978V packaged DNA at levels approximately 28.6 and 24.0 ng/μg protein, respectively, values comparable to or exceeding those of the functional donor A2485 at approximately 13.6 ng/μg, directly ruling out packaging deficiency as the basis for their transfer failure. Among nAB strains, *A. junii* and *A. pittii* packaged substantially less DNA at approximately 4.5–4.8 ng/μg, suggesting a host-dependent constraint on vesicular cargo loading. However, *A. nosocomialis* LMG10619 packaged DNA at levels approaching those of *A. baumannii* at approximately 11.7 ng/μg, yet also failed as a donor, effectively decoupling packaging quantity from transfer competence and demonstrating that reduced cargo loading alone is insufficient to account for the observed species barrier (47, 48).

Transfer was further characterized by a striking non-reciprocity. Recipient strains 17978E and 17978V, despite stably carrying *p*A2485, were unable to retransmit the plasmid under identical experimental conditions, confirming that transfer competence is not a property conferred by plasmid possession alone. Successful electroporation of *p*A2485 into several nAB strains confirmed their biological capacity to replicate and maintain the plasmid, yet OMVs from these strains consistently failed to deliver *bla*_OXA-72_ to either nAB or *A. baumannii* recipients. Taken together, these findings converge on a post-packaging impediment, most plausibly related to OMV surface composition, recipient cell recognition, or membrane fusion competence, as the primary determinant of donor specificity.

In light of these observations, we propose a host licensing model in which productive OMV-mediated plasmid transfer requires donor-specific cellular factors, such as envelope proteins or surface ligands, that license plasmid encapsulation and delivery and thereby restrict dissemination to defined host lineages. This framework is mechanistically distinct from conjugation, which depends on plasmid-encoded transfer machinery, and from natural transformation, which relies on host competence systems. It should be noted that our experiments employed *p*A2485, a plasmid of *A. baumannii* origin, and we therefore cannot exclude the possibility that nAB species may effectively transfer their own native plasmids via OMVs. Future studies utilizing species-specific plasmids will be necessary to fully resolve the relative contributions of plasmid identity and host-specific OMV machinery to this process.

The restricted nature of OMV-mediated transfer carries important epidemiological implications, offering a mechanistic explanation for why *bla*_OXA-72_ remains largely confined to *A. baumannii* in China despite the global distribution of conserved plasmid backbones. Such host-specific barriers may limit resistance dissemination to particular lineages while simultaneously facilitating localized adaptation within clinical niches. Identifying the molecular determinants of host licensing, whether associated with vesicle biogenesis, cargo selection, or recipient recognition, will be an important priority. Targeting these factors may ultimately offer novel strategies to impede plasmid-mediated resistance spread in hospital settings.

## SUPPLEMENTAL MATERIAL

Supplemental material msphere.00311-26-s0001.pdf Fig. S1 to S5; Tables S1 to S5.

ASM does not own the copyrights to Supplemental Material that may be linked to, or accessed through, an article. The authors have granted ASM a non-exclusive, world-wide license to publish the Supplemental Material files. Please contact the corresponding author directly for reuse.
