# Supplementary material for: Strain-specific outer membrane vesicle-mediated transfer of a blaOXA-24-like plasmid in Acinetobacter baumannii restricts cross-species dissemination
Source: mSphere. 2026 Aug 20;11(9):e00311-26. doi: 10.1128/msphere.00311-26 (PMC13621841; doi:10.1128/msphere.00311-26)
Supplement: Supplementary file 1 [file msphere.00311-26-s0001.pdf]

Supplementary information
This file contains figures S1-S5, and tables S1-S5.

Fig. S1: Genetic structures upstream and downstream of the *bla*$_{OXA-72}$ gene. 2
Fig. S2: Comparative genomic analysis of plasmid p352 and related *Acinetobacter* plasmids. 2
Fig. S3: Chromosomal resistome of strain A2485. 3
Fig. S4: PFGE comparison of *Acinetobacter baumannii* strains 17978V, 17978, and A2485. 4
Fig. S5. PCR analysis confirmed the presence of pA2485 within OMVs. 5
Table S1. Primer sequences used for PCR-based gap closure. 5
Table S2. Resistance Gene Analysis Profile of A2485. 8
Table S3. BLAST-based identification of known chromosomal resistance islands in *Acinetobacter baumannii* A2485. 9
Table S4: Information on the transposon of A2485. 10
Table S5: Transformation assay of A2485-derived OMVs and supernatant after 0.125% Triton X-100 treatment. 11

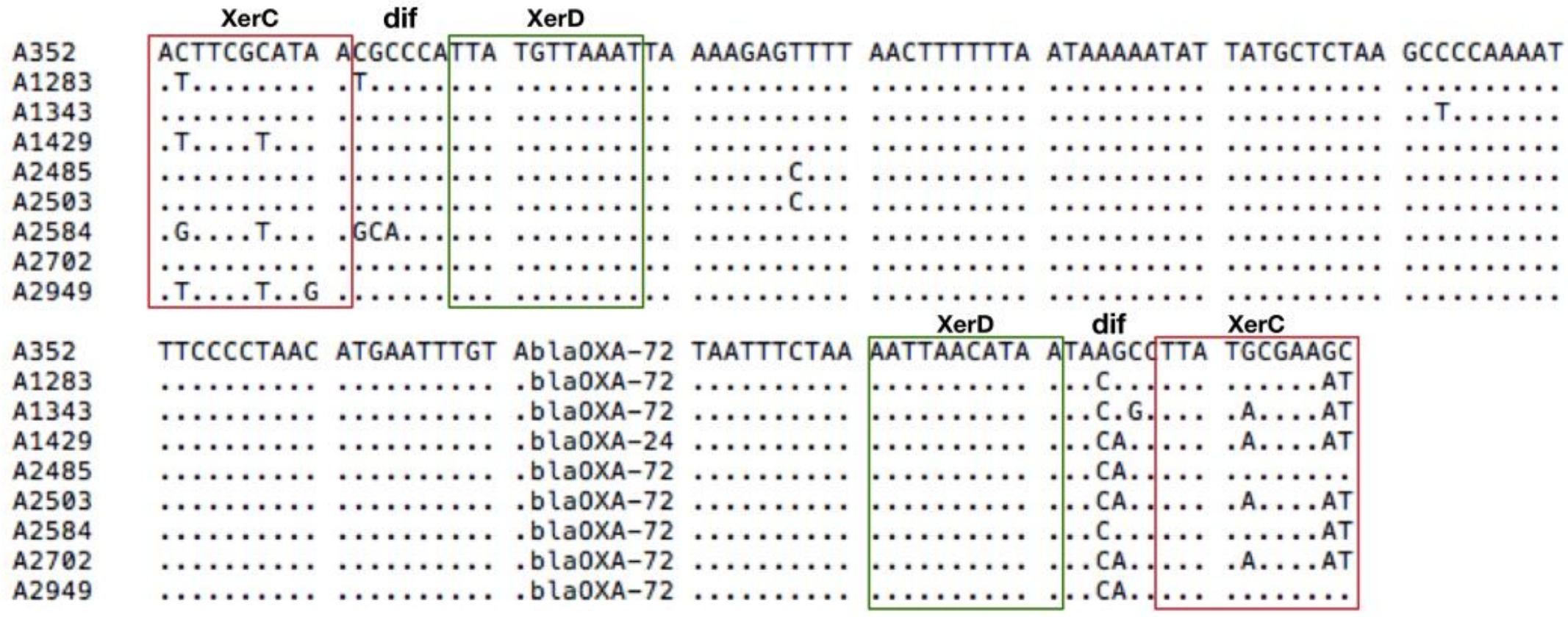

Fig. S1: Genetic structures upstream and downstream of the *bla*$_{OXA-72}$ gene.

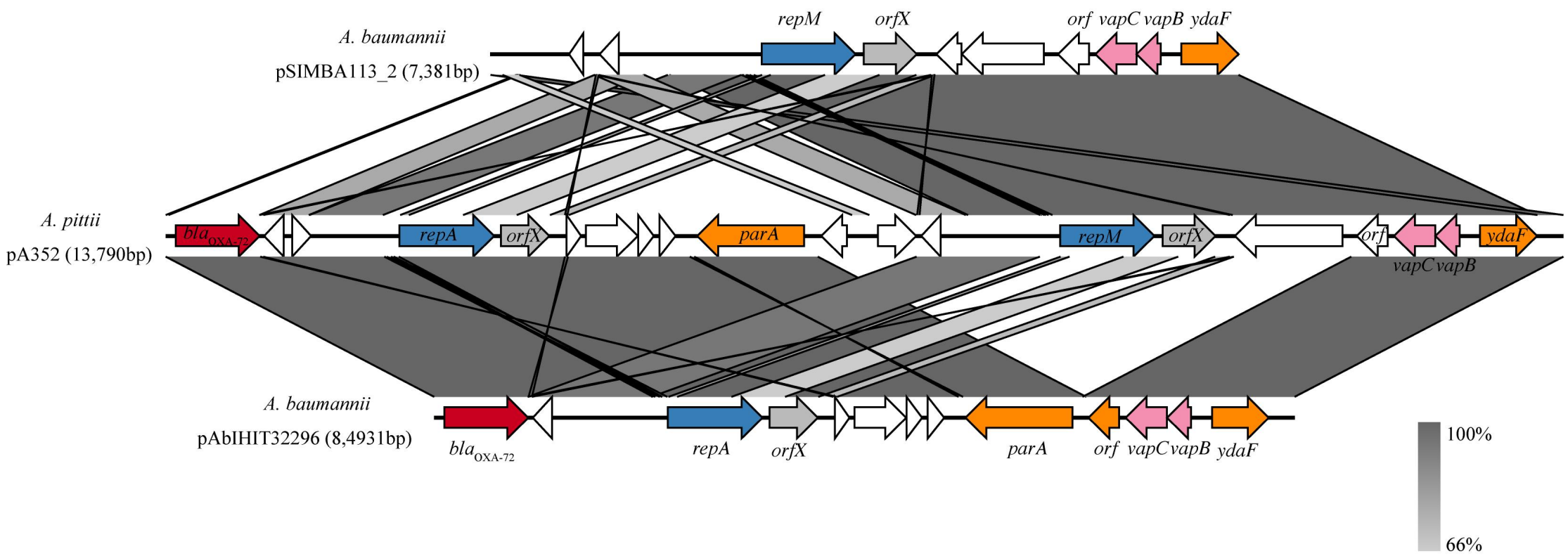

Fig. S2: Comparative genomic analysis of plasmid p352 and related *Acinetobacter* plasmids.

Linear alignment showing that plasmid *p*352 is a cointegrate structure derived from *A. baumannii* plasmids pAbIHIT32296 and *p*SIMBA113_2. The first half of p352 displays near-complete identity with *p*AbIHIT32296, while the second half shares high similarity with *p*SIMBA113_2. Arrows indicate open reading frames and their orientations, with key backbone genes (e.g., *repA*, *repM*, *parA*, *vapC/vapB*, *ydaF*) highlighted. Shaded regions represent sequence homology, with darker shading indicating higher nucleotide identity. Genetic structures upstream and downstream of the *bla*$_{OXA-72}$ gene.

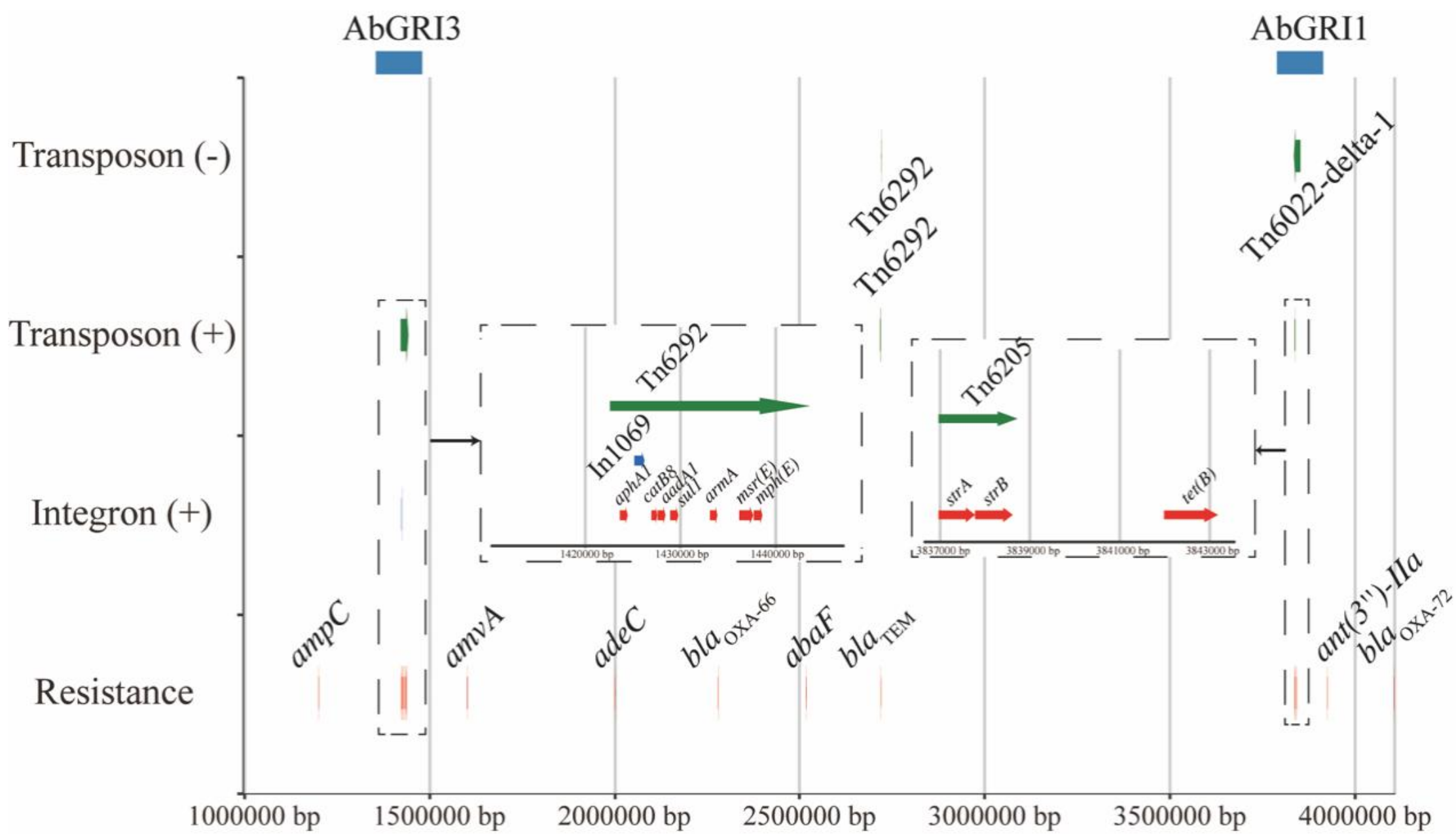

Fig. S3: Chromosomal resistome of strain A2485.

Intrinsic β-lactamase loci include *bla*$_{OXA-66}$, encoding an OXA-51-like carbapenemase, and *bla*$_{ADC-30}$, encoding an AmpC-type cephalosporinase. The latter is associated with an upstream ISAba1 insertion, consistent with an ISAba1-activated *bla*$_{ADC}$ expression module. Several acquired resistance genes are located within chromosomal resistance islands. An AbGRI1-1-like region, detected with 100% coverage and 100% nucleotide identity, carries *tet(B)*, *aph(3'')-Ib/strA*, and *aph(6)-Id/strB*. An AbGRI3-2-like region, detected with 89.7% coverage and 100% nucleotide identity, carries *armA*, *msr(E)*, *mph(E)*, *catB8*, *sul1*, *aadA1*, *aacA4*, and *aph(3')-Ia/aphA1b*. Other chromosomally encoded resistance-associated determinants include the MFS transporters *amvA* and *abaF*, and the RND efflux component *adeC*. The blue boxes indicate the positions of AbGRI1 and AbGRI3 regions.

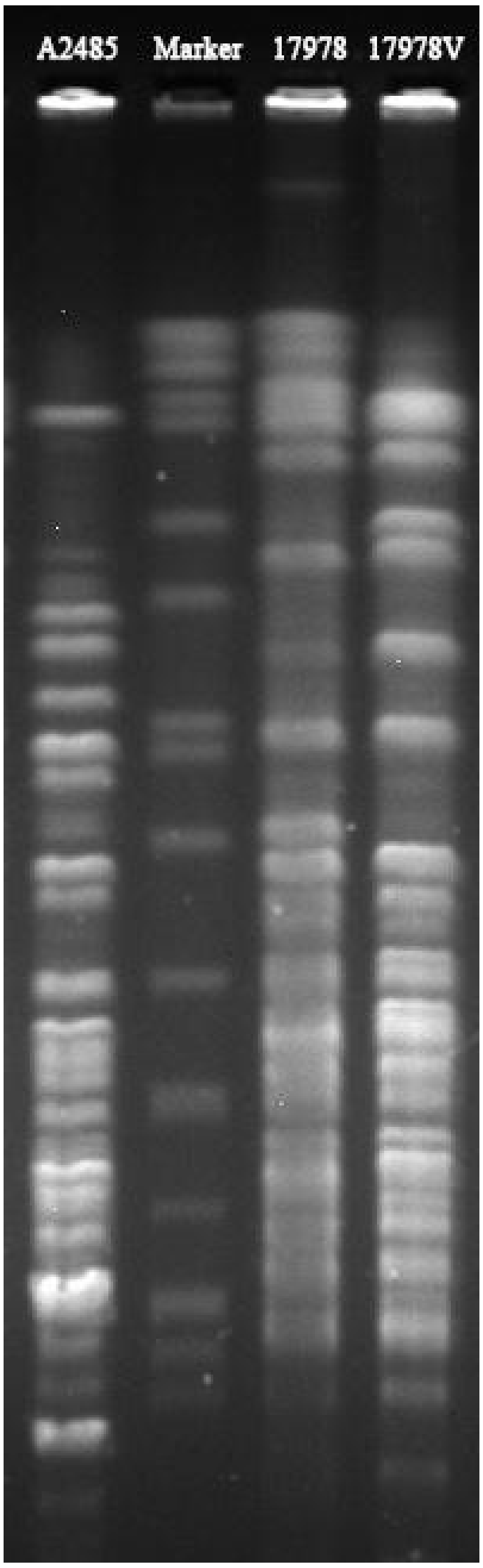

Fig. S4: PFGE comparison of *Acinetobacter baumannii* strains 17978V, 17978, and A2485.

Marker: Salmonella enterica serotype Braenderup strain H9812.

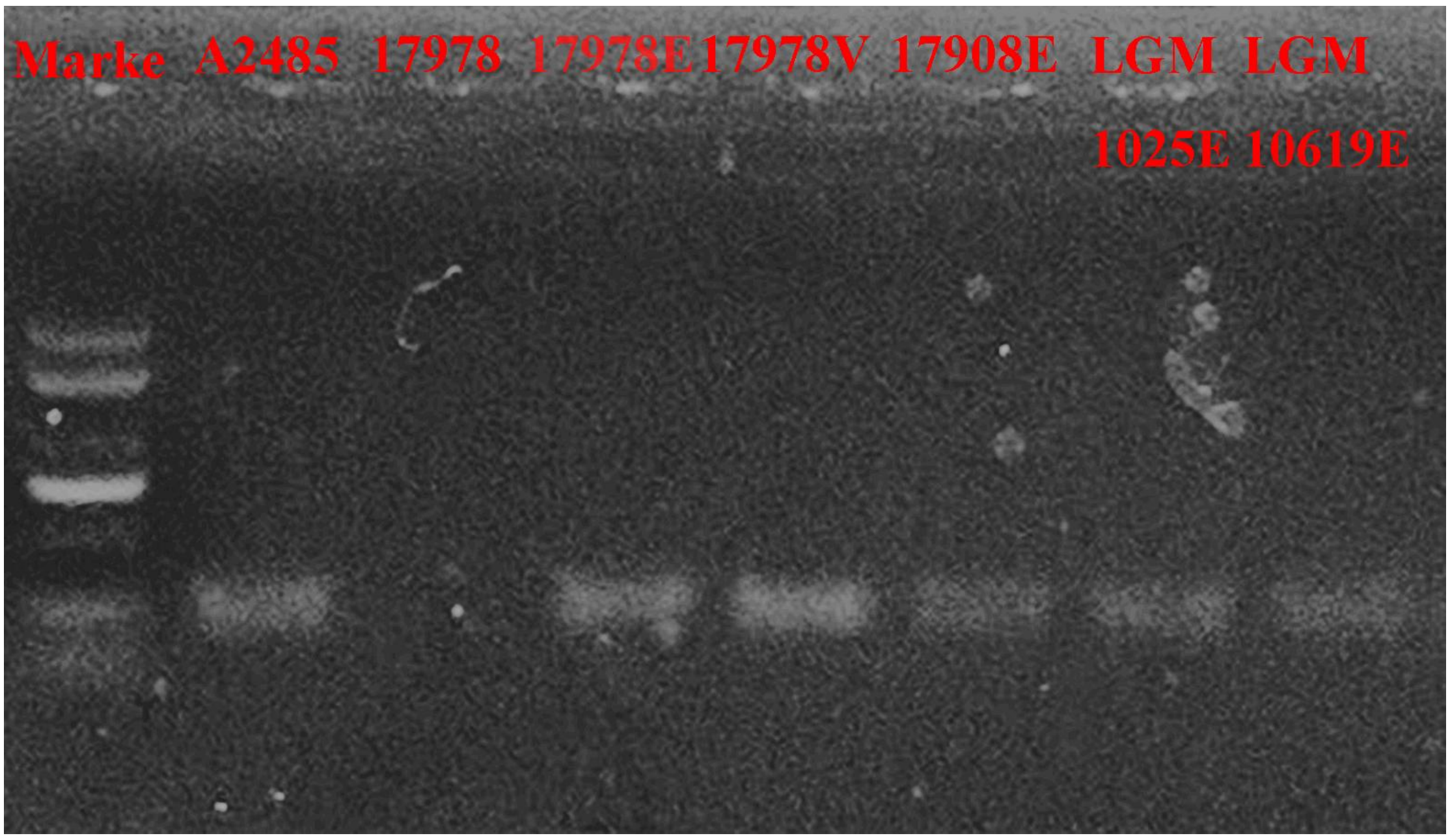

Fig. S5. PCR analysis confirmed the presence of pA2485 within OMVs.

Table S1. Primer sequences used for PCR-based gap closure.

Primer	Sequence (5' to 3')
1429-walking 1	GTCTACTCTAACTGGGTAATTG
1429-walking 2	GCTCTTCAAAACTCTTCGT
352-walking 1	TTCGGATGTTTTCCTGAC
352-walking 2	ACTTAATTTGGGGTCGAT
352-walking 3	CCATCAATGACACTCCCT
352-walking 4	TGGCCGATATAAGCTCTA
352-walking 5	CTTGCCTGTAAAATAGCC
352-walking 6	GTAACATCCATTCCCCAT
352-walking 7	CATGGTTCTTAATTCGGTAC
352-walking 8	TGAGAGTATTTCGCCATTT
352-walking 9	ACAGATGCTCAACGCCAC
352-walking 10	TGATGCTGCAACCTTAGA
352-walking 11	AGCACCAGTCCTACGTCA
352-walking 12	CCTTGCACATAACCGATT
352-walking 13	ACCCGCTTTACTTCTTTC
352-walking 14	CTCGGTTAATAATGTGGATA
352-walking 15	AATGGAGATTTAGATTGGGATG
352-walking 16	TGATGCAAAAGAGCGTGA
352-walking 17	GATGATGCTGCAACCTTA
352-walking 18	ACTCGCAAACCATTCCTT
352-walking 19	GTAGGCGGCATGTTTGTT
352-walking 20	GGACTGGTGCTTGGTTTT
1343-walking 1	AGGGCAAAATCTAAAACATACG

1343-walking 2	TTGGGGAAGATGGGCTAT
1343-walking 3	GTTGATACTGATTCAGCCACT
1343-walking 4	CTCGGTCTAGGAACAAATTT
1343-walking 5	TATCGACCCCAAATTAAG
1343-walking 6	AATGCGGATGAACTACAG
1343-walking 7	TTTCCCCTAACATGAATTTG
1343-walking 8	AGGTTAATCCTGGGCTTT
1343-walking 9	CTATCCGTGTTTTTGGTG
1343-walking 10	ACTTTCCATGGGTTTTTC
1343-walking 11	TTTGGGTCTAAGTGAATGA
1343-walking 12	TTAGGAAGCGATAAGTTGA
2584-walking 1	TGTACTTCGCATAACC
2584-walking 2	ATGATGTCCCAAGAAG
2584-walking 3	ATGCCATTGCCTCACC
2584-walking 4	GCTCTGCCGTTTGTCTG
2584-walking 5	ATGCCTTAGCCTTGTGATG
2584-walking 6	CGATAGCCTGTTTTTGAGC
2584-walking 7	TTGCTTACGGACAAACTCT
2584-walking 8	TGTCCAGGTTCTCCACTTT
2584-walking 9	CAAAGAATAGCCGTGGAAC
2584-walking 10	CATTAGGAAGCGATAAGTTG
2584-walking 11	CTCCCCTTAGTGGTTGTTAT
2584-walking 12	TGACATTATCGGAGACACTG
2584-walking 13	TTCAAGCGGGTAATATGC
2584-walking 14	GATGATACTCGGCGTAAAAT
2702-walking 1	ATCAGCGGGGTTGTTT
2702-walking 2	TGCAGCTCACAGGGTC
2702-walking 3	GCAAATTACGCCCTGGAC
2702-walking 4	TTGGGATCTCGTTTAGTCG
2702-walking 5	ACAGCGTTTGATCATGC
2702-walking 6	AAGCCCAGTTAGAAAAGC
2702-walking 7	GGACGCGCTGCTAAAT
2702-walking 8	AATGGTCTGGGGCACT
2702-walking 9	ACGTCATTTATTCGCAACT
2702-walking 10	CGAAAGCATGGCGTAC
2702-walking 11	GACAGGGCAACAAGTAAT
2702-walking 12	ATGGCGAATGGAAAGA
2702-walking 13	GGTGGAGCAAGCTAATG
2702-walking 14	CAAACCTCTATGGAATGACT
2702-walking 15	CTATTCGCAAAATCCCT
2702-walking 16	GTGCAAGAGCATTACCAT

2485-walking 1	CGGACTTCGTTCTATGC
2485-walking 2	CAGTTACCATTTCAGCGT
2503-walking 1	GGAATGGATGTTTACTCCAC
2503-walking 2	GACACCACAACAAGACAATG
2503-walking 3	GTGTAATAAAGCCACTCCTC
2503-walking 4	TTAAGCAGCAGGCAGATC
2587-walking 1	TCTAGCCCGATTAAAGCA
2587-walking 2	ATTGGCGAAAGATGACA
2587-walking 3	CGAAGCGAAAACCAAG
2587-walking 4	GACCCAACGACAGTGAA
2587-walking 5	GCCTTAGCCTTGTGATGG
2587-walking 6	TTAGGACAGGGCATCACG
2587-walking 7	GGTAATCGGTTATGTGCA
2587-walking 8	GATATTTTGGCTGCTTGA
2587-walking 9	ACCATAGGTGCTAAGATT
2587-walking 10	TTTTAGATACGAAGTGGC
2587-walking 11	ATTAGATATGAAGGGCAAGA
2587-walking 12	ACAAAATGAAACCGTGGA
1283-walking 1	CATGGGGTTGAAACGTGT
1283-walking 2	GAAAGATGGGGGAAAAGC
1283-walking 3	ATTGCCTAGCTTCAAGTATGG
1283-walking 4	CAGTTTTAAGCACTCCCCTCT
1283-walking 5	TCCTTTTCGTAAATAGCG
1283-walking 6	CCTGAAAATACTCGTAGCC
1283-walking 7	ATCAAGAACAGTCGCAAGC
1283-walking 8	ATCAAGGGCAAGGTATCA
2949-walking 1	TTACCCAACGGCTATGAG
2949-walking 2	GTCTACGAGCGAAGCACC
2949-walking 3	CGCTGCGATTAGTAGTTT
2949-walking 4	TTGTGCTAAGGGCTGTAA
2949-walking 5	AGAATCGCTAAAGAAATGAG
2949-walking 6	TGTCTACCAAGGTGAGGC
2949-walking 7	TGGAATGTCTATTTTGATGG
2949-walking 8	TTTTGAACACGGTTTACTC

Table S2. Resistance Gene Analysis Profile of A2485.

SEQUENCE	START	END	STRAND	GENE	COVERAGE	GAPS	%COVERAGE	%IDENTITY	DATABASE	ACCESSION	PRODUCT
Chromosome	1200393	1201544	minus	*blaADC-30*	1-1152/1152	0	100	100	resDB	NG_048652.1	CEPHALOSPORIN
Chromosome	1423647	1424462	plus	*aph(3')-Ia*	1-816/816	0	100	99.88	resDB	NG_047431.1	KANAMYCIN
Chromosome	1426956	1427588	plus	*catB8*	1-633/633	0	100	100	resDB	NG_047616.1	CHLORAMPHENICOL
Chromosome	1427646	1428437	plus	*aadA1*	1-792/792	0	100	100	resDB	JQ414041	SPECTINOMYCIN/STREPTOMYCIN
Chromosome	1428942	1429781	plus	*sul1*	1-840/840	0	100	100	resDB	NG_048082.1	SULFONAMIDE
Chromosome	1433126	1433899	plus	*armA*	1-774/774	0	100	100	resDB	NG_047476.1	GENTAMICIN
Chromosome	1436198	1437673	plus	*msr(E)*	1-1476/1476	0	100	100	resDB	NG_048007.1	MACROLIDE
Chromosome	1437729	1438613	plus	*mph(E)*	1-885/885	0	100	100	resDB	NG_064660.1	MACROLIDE
Chromosome	1602612	1604090	plus	*amvA*	1-1479/1479	0	100	97.16	resDB	FJ911554.1	EFFLUX
Chromosome	2001444	2002841	plus	*adeC*	1-1398/1398	0	100	100	resDB	AF370885.1	EFFLUX
Chromosome	2279887	2280711	plus	*blaOXA-66*	1-825/825	0	100	100	resDB	NG_049806.1	BETA-LACTAM

Chromosome	2517206	2518495	plus	*abaF*	1-1290/1290	0	100	97.75	resDB	CP000521.1	FOSFOMYCIN
Chromosome	2719274	2720134	minus	*blaTEM-1D_1*	1-861/861	0	100	100	resDB	AF188200	AMOXICILLIN
Chromosome	3836966	3837779	plus	*aph(3'')-Ib*	15-828/828	0	98.31	99.88	resDB	NG_056002.2	STREPTOMYCIN
Chromosome	3837779	3838615	plus	*aph(6)-Id*	1-837/837	0	100	100	resDB	NG_047464.1	STREPTOMYCIN
Chromosome	3841988	3843187	plus	*tet(B)*	1-1200/1206	0	99.5	100	resDB	NG_048161.1	TETRACYCLINE
Chromosome	3925186	3925974	minus	*ant(3'')-IIa*	1-789/789	0	100	98.61	resDB	NG_054646.1	STREPTOMYCIN
Plasmid	12780	13607	plus	*blaOXA-72*	1-828/828	0	100	100	resDB	NG_049813.1	BETA-LACTAM

Table S3. BLAST-based identification of known chromosomal resistance islands in *Acinetobacter baumannii* A2485.

Strain	Locus type	Closest known variant	Overall coverage (%)	Identity (%)	Reference island length (bp)	Position in A2485 sequence	Position in reference island	Strand	Resistance genes assigned to the region	Interpretation
A24	AbG	AbGR	100	100	17630	57040..7466	1..17630	-	tetA(B)/tet(B), tetR(B),	Complete AbGRI1-1-like

85	RI1	I1-1				9			strA [aph(3'')-Ib], strB [aph(6)-Id]	chromosomal resistance island detected by BLAST-based profiling.
A24 85	AbG RI3	AbGR I3-2	89.7 32	100	20959	contig00035 : 1..17654; contig00060 : 1..1153	contig00035: 711..18364; contig00060: 18963..20115	+	aphA1b [aph(3')-Ia], mph(E), msr(E), armA, sul1, qacEdelta1, aadA1, catB8, aacA4	AbGRI3-2-like chromosomal resistance island detected across two contigs; matched segments show 100% nucleotide identity.

Table S4: Information on the transposon of A2485.

QUERY	TRANSPOSON TYPE|ACCESSION	%IDENTITY	LENGTH	MISMATCH	GAPOPEN	QUERY START	QUERY END	SUBJECT START	SUBJECT END	EVALUE	BITSCORE	%COVERAGE
1	Tn6292	99.64	823	3	0	1422623	1423445	443	1265	0	1471	65.01
1	Tn6279|KT317075	100	20960	0	0	1422626	1443585	1	20960	0	37799	100
1	Tn6292	99.76	820	2	0	1424512	1425331	446	1265	0	1471	64.77
1	Tn6292	99.76	822	2	0	1442763	1443584	444	1265	0	1474	64.93
1	Tn6292	100	820	0	0	2717370	2718189	446	1265	0	1480	64.77
1	Tn6292	99.64	830	3	0	2718426	271925	436	1265	0	1483	65.56

							5					
1	Tn6292	100	820	0	0	2720834	2721653	1265	446	0	1480	64.77
1	Tn6166	100	17631	0	0	3834705	3852335	17631	1	0	31796	100
1	Tn6205|CP003505	100	1756	0	0	3836966	3838721	1006	2761	0	3168	63.58
1	Tn6022-delta-1|JN247441	100	9149	0	0	3843187	3852335	9149	1	0	16500	100

Table S5: Transformation assay of A2485-derived OMVs and supernatant after 0.125% Triton X-100 treatment.

Donor Type[a]	Recipient Strain	Recipient Species	Transformation Efficiency
OMVs + Triton X-100	ATCC17978	*A. baumannii*	0
OMVs + Triton X-100	ATCC11171	*A. guillouiae*	0
OMVs + Triton X-100	ATCC15309	*A. lwoffii*	0
OMVs + Triton X-100	ATCC17906	*A. haemolyticus*	0
OMVs + Triton X-100	ATCC17908	*A. junii*	0
OMVs + Triton X-100	ATCC17909	*A. johnsonii*	0
OMVs + Triton X-100	ATCC17924	*A. bereziniae*	0
OMVs + Triton X-100	ATCC23055	*A. calcoaceticus*	0
OMVs + Triton X-100	ATCC31012	*A. venetianus*	0
OMVs + Triton X-100	ATCC43998	*A. radioresistens*	0
OMVs + Triton X-100	A354	*A. seifertii*	0

OMVs + Triton X-100	LMG1035	*A. pittii*	0
OMVs + Triton X-100	LMG10619	*A. nosocomialis*	0
OMVs + Triton X-100	DSM18516	*A. brisouii*	0
Supernatant + Triton X-100	ATCC17978	*A. baumannii*	0
Supernatant + Triton X-100	ATCC11171	*A. guillouiae*	0
Supernatant + Triton X-100	ATCC15309	*A. lwoffii*	0
Supernatant + Triton X-100	ATCC17906	*A. haemolyticus*	0
Supernatant + Triton X-100	ATCC17908	*A. junii*	0
Supernatant + Triton X-100	ATCC17909	*A. johnsonii*	0
Supernatant + Triton X-100	ATCC17924	*A. bereziniae*	0
Supernatant + Triton X-100	ATCC23055	*A. calcoaceticus*	0
Supernatant + Triton X-100	ATCC31012	*A. venetianus*	0
Supernatant + Triton X-100	ATCC43998	*A. radioresistens*	0
Supernatant + Triton X-100	A354	*A. seifertii*	0
Supernatant + Triton X-100	LMG1035	*A. pittii*	0
Supernatant + Triton X-100	LMG10619	*A. nosocomialis*	0
Supernatant + Triton X-100	DSM18516	*A. brisouii*	0

[a]OMVs and supernatant pretreated with 0.125% Triton X-100 at 37 °C for 30 min.
